# Sustainable Production of the Cyanophycin Biopolymer in Tobacco in the Greenhouse and Field

**DOI:** 10.3389/fbioe.2022.896863

**Published:** 2022-06-13

**Authors:** Jana Huckauf, Boudewijn P. Brandt, Carlos Dezar, Henrik Nausch, Antoniya Hauerwaas, Ursula Weisenfeld, Ossama Elshiewy, Melina Rua, Jeroen Hugenholtz, Justus Wesseler, Kutay Cingiz, Inge Broer

**Affiliations:** ^1^ Agrobiotechnology, University of Rostock, Rostock, Germany; ^2^ Bioceres S.A., Rosario, Argentina; ^3^ Institute of Management and Organisation (IMO), Leuphana University Lüneburg, Lüneburg, Germany; ^4^ Wageningen Food and Biobased Research, Wageningen, Netherlands; ^5^ Agricultural Economics and Rural Policy, Wageningen University, Wageningen, Netherlands

**Keywords:** cyanophycin, plant made industrials, sustainable production, field trial, isolation, cost benefit analysis, market analysis, consumer acceptance

## Abstract

The production of biodegradable polymers as coproducts of other commercially relevant plant components can be a sustainable strategy to decrease the carbon footprint and increase the commercial value of a plant. The biodegradable polymer cyanophycin granular polypeptide (CGP) was expressed in the leaves of a commercial tobacco variety, whose seeds can serve as a source for biofuel and feed. In T0 generation in the greenhouse, up to 11% of the leaf dry weight corresponded to the CGP. In T1 generation, the maximum content decreased to approximately 4% dw, both in the greenhouse and first field trial. In the field, a maximum harvest of 4 g CGP/plant could be obtained. Independent of the CGP content, most transgenic plants exhibited a slight yield penalty in the leaf biomass, especially under stress conditions in greenhouse and field trials. After the harvest, the leaves were either Sun dried or ensiled. The resulting material was used to evaluate the extraction of CGP compared to that in the laboratory protocol. The farm-level analysis indicates that the extraction of CGP from tobacco plants can provide alternative income opportunities for tobacco farmers. The CGP yield/ha indicates that the CGP production in plants can be economically feasible depending on the cultivation and extraction costs. Moreover, we analyzed the consumer acceptance of potential applications associated with GM tobacco in four European countries (Germany, Finland, Italy and the Netherlands) and found unexpectedly high acceptance.

## Introduction

The sustainable production of petroleum-based compounds such as fine chemicals is a key challenge in the modern society. Biotechnology provides tools for the high-yield production of these compounds. Generally, production is performed through microbial, plant or animal cell cultures, and this strategy is especially suitable for sterile production (Twyman et al., 2003). Moreover, compounds with a high demand but low value are also targets of biotechnological approaches. Here, cell cultures are too expensive to maintain, prone to contamination and inflexible to rapidly changing demands (Twyman et al., 2003). Transgenic plants grown in the field can serve as cost-effective alternatives. Several such products are already available in the market, as reviewed in (Ricroch et al., 2022). Field-grown tobacco exhibits a high yield of bacterial cellulase in the chloroplasts (Schmidt et al., 2019). Moreover, plants can synthesize polyhydroxyalkanoates with properties similar to polyacrylates or other synthetic polymers (such as polyhydroxybutyrate (PHB) (Anderson and Dawes, 1990; Poirier, 2002; Börnke and Broer, 2010) or the polydisperse biopolymer cyanophycin granular polypeptide (CGP) (Borzi, 1887). The transition from fossil to plant-based production can decrease the energy consumption. Such production processes exhibit a decreased cost, and the adverse influences of the use of fossil raw materials can be alleviated (Mooibroek et al., 2007). They can also facilitate the transition toward sustainable food systems, as discussed at the UN Food System Summit in 2021 (Trigo et al., 2021).

However, in all of these cases, the return on investment occurs solely via the market, depending on the value of the transgene–encoded compound and changes in the production costs. The manufacturing of valuable compounds as coproducts for plant-made industrials may be a cost-effective and environmentally friendly alternative. In this case, production does not depend on additional arable land or energy. The compound can be purified from the leftovers after isolation of the primary product. The biopolymer CGP has been produced as such a coproduct for starch potato (Hühns et al., 2008; Schmidt et al., 2017). CGP is a polydisperse (25–125 kDa) polymer consisting of mainly L-aspartic acid (Asp) and L-arginine (Arg), synthesized by a cyanophycin synthetase. Many cyanobacteria and several nonphotosynthetic bacteria produce the enzyme encoded by a *cph*A gene (Krehenbrink et al., 2002; Ziegler et al., 2002).

CGP, Asp and Arg have been applied as fine chemicals (Mooibroek et al., 2007). In particular, Arg-Asp dipeptides can substitute free Arg and Asp in several food and feed additives since their bioavailability is superior to that of free amino acids. Various commercial products of these forms are already available in the market (Sallam and Steinbüchel, 2010). In addition, various potential applications in the nonfood/feed industry have been suggested. By removing Arg from CGP, poly-asp can be produced, which has applications ranging from water-softening or detergent applications to applications in the paper, building material, petroleum, cosmetics, or leather industries and dispersant-based applications (Schwamborn, 1996; Schwamborn, 1998; Elbahloul et al., 2005; Mooibroek et al., 2007; Börnke and Broer, 2010). Moreover, Asp can be converted to acrylonitrile, which is widely applied in acrylic fibers, nitrile rubbers, and carbon fibers.

Arg can be converted to 1,4-butanediamine and urea. At present, 1,4-butanediamine is used to produce nylon-4,6 (Mooibroek et al., 2007). The material is primarily used in applications that require high thermal stability and tensile strength, such as engineering materials and heavy machinery. The remaining urea can also be applied in industries.

The biopolymers extracted from CGP are highly valued. As reviewed in Sallam and Steinbüchel, (2010), dipeptide L-alanyl-L-glutamine, which is used as an infusion to treat patients with immunosuppression, postoperative complications or malnutrition, is offered by Mediatech Inc. (Manassas, Virginia, United States) for €4,732/kg. Salam and Steinbüchel assumed that CGP dipeptides are at least as effective as these products in several fields of application. Hence, the researchers predicted that CGP dipeptides can reach a market price of over €3,000/kg. Accordingly, plant-based CGP has considerable market potential, especially in Argentina and other Latin American countries with a substantial amount of tobacco production, and can facilitate the development of a sustainable bioeconomy (Trigo et al., 2021). Nevertheless, this product must compete with CGP derived from cyanobacteria (Lippi et al., 2018), or synthesized from *Escherichia coli* or yeast (Sallam et al., 2011).which all represents active areas of research Certain commercial success has been achieved with synthesis based on *Escherichia coli* (Kwiatos and Steinbüchel, 2021).

The expression of the gene *cph*ATe from the cyanobacterium *Thermosynechococcus elongatus* BP-1 under the control of the cauliflower mosaic virus (CaMV) 35S promoter and terminator in transgenic tobacco Petit Havana SR1 plants resulted in up to 1.0% of CGP dry weight (dw) (Neumann et al., 2005). Phenotypic damage caused by the production of CGP in the cytosol was eliminated via the plastid location of the synthetase. This modification led to CGP contents of up to 1.7 and 6.8% dw in the T0 and T2 descendants, respectively, without detectable stress symptoms (Hühns et al., 2008). Moreover, CGP-producing potato plants were analyzed in 6 years of field trials (Schmidt et al., 2017). Without additional fertilization or other inputs, the plants produced up to 4 μg/mg dw (0.4% dw) of CGP in the tubers without a significant reduction in the starch content. However, the tuber size was significantly decreased. Although the number of tubers increased, the overall yield was less compared to near isogenic control (NIC) (Schmidt et al., 2017).

The production of CGP in the commercial tobacco cultivars Badischer Geudertheimer (BG) and Virginia Golta (VG) in the greenhouse was noted to be a highly effective process. In the F1 hybrids (max. 9.4% CGP dw) and T0 transformants (max. 8.8% CGP dw), the CGP content was significantly higher than that achieved through Petit Havana SR1 transformants. In the greenhouse, no significant yield penalty was observed (Nausch et al., 2016). In addition, the cytosolic expression of a cyanophycinase, encoded by the gene *cph*EPa, in CGP-producing tobacco led to the formation of stable Arg-Asp dipeptides after cell disruption (Nausch and Broer, 2017; Ponndorf et al., 2017; Nausch et al., 2020). Hence, commercial tobacco varieties may be a suitable production platform for CGP.

Moreover, tobacco can be used to produce CGP as a coproduct of a number of other products in addition to extracted nicotine. Recently, tobacco has received renewed attention as a bioenergy crop (Gowtham Rajan et al., 2021). Tobacco plants can provide substantial amounts of oil stored in the seeds (Grisan et al., 2016) and yield fermentable sugars for bioethanol or biogas (Schneider et al., 2021). Tobacco oil has been successfully converted to biodiesel that can satisfy the European standard (Usta et al., 2011). The biomass remaining after extracting CGP from the plant can be applied in the biobased economy.

The possibility of storing the harvest is key to cost-effective processing because the harvest can be processed over a prolonged period in a facility with a smaller maximum capacity and lower investment cost. CGP-containing tobacco leaves can be stored in a dried form or as silage without loss of CGP (Nausch et al., 2016). Isolation of plant-made CGP on a small scale has been reported for tobacco and potato, achieving purities of 58–90%, respectively (Neubauer et al., 2012). This method is based on acid extraction on lyophilized starting material. Freeze-drying is an efficient technique to expand plant cells for extraction; however, the process is highly energy intensive to be applied at a large scale (Guldhe et al., 2014). In addition, the common extraction protocol applies 0.1 M HCl, which is corrosive and requires specially adapted machinery. In this study, we evaluate the possibility of macerating dried and ensiled tobacco leaf material.at pH 5, omitting low pH conditions in the expensive equipment, followed by extraction at pH 1, which can be performed in a simple tank. The findings can facilitate the processing of farm-scale quantities of CGP-containing tobacco.

The approval to cultivate GM crops in Europe is difficult to obtain (Sparrow et al., 2013; Smart et al., 2015). Many companies attempt to set bases in other countries, such as Argentina or the United States, where the approval process to cultivate GM crops is considered to be transparent, less complicated, and less expensive than that in Europe (Whelan and Lema, 2015; Eriksson et al., 2019). Nonfood/feed products derived from transgenic plants do not require any additional GMO labeling in the EU (Regulation (EC) No. 1829/2003, (Wesseler and Kalaitzandonakes, 2019)). Nevertheless, the import and processing of CGP from transgenic tobacco require approval. These procedures and costs can be avoided by processing CGP into the final product in the home country and exporting the final product to the EU, similar to the export of textiles derived from GM cotton. Nevertheless, a market for GM-free labeled textiles has emerged for cotton. Similar voluntary labeling has been observed for a number of food and nonfood products in the EU, with substantial implications for the marketability of GM food/feed and nonfood/feed products owing to consumer responses (Castellari et al., 2018). Hence, any product isolated from genetically modified plants (GMP) to be offered in a consumer market requires consumer acceptance. Therefore, we conducted choice experiments for two hypothetical applications mimicking consumers’ shopping and consumption patterns: food wrapping and cosmetics.

To clarify whether the production of CGP can be a sustainable alternative, we conducted field trials using several independent transgenic events from the tobacco cultivar BG in Argentina, tested the isolation from storable materials and evaluated the production and potential commercial value of CGP products along with the consumer acceptance for these products in four European countries.

## Materials and Methods

### Tobacco Transformation

**FIGURE 1:**
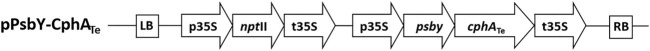
Map of the nuclear transformation vector for constitutive, plastidic expression of the cyanophycin synthetase. *cphA*Te: cyanobacterial coding region of theCP-synthetasefrom *T.elongatus* BP-1, *psby*:peptide of the integral protein of photosystemII, p35S:constitutive cauliflower mosaic virus (CaMV) 35S promoter; t35S:CaMV terminator, *npt*II:coding region of neomycin phosphotransferase gene, LB and RB, left and right borders of *A.tumefaciens* binary vector (Hühns et al., 2008).

The vector pPsbY*-Cph*A_Te ’_ (Figure 1) (Hühns et al., 2008) was introduced into tobacco by the *Agrobacterium tumefaciens* strain LBA4404 through leaf disc transformation, with reference to an existing study (Wohlleben et al., 1988). Regenerated plants were analyzed through PCR, transferred to an *in vitro* cultivation platform and subsequently transferred to soil in the greenhouse. *In vitro* cultivation was performed in LS medium in a growth chamber at 21°C for a light/dark period of 16/8 h.

### Greenhouse Cultivation

In Rostock (Germany), transgenic individuals (T0 transformants and T1 descendants) were transferred from *in vitro* culture 4 weeks after the last subculture directly into 3.5 L pots containing peat soil (Stender AG, Schramberg, Germany). Plants were fertilized twice a week using 0.2% Hakaphos Blue (Hermann Meyer KG, Rellingen Germany). The phenotypes were assessed after 4 and 12 weeks. Leaf samples were obtained 12 weeks after potting. All leaves of each single plant were harvested, freeze dried and powdered. Subsamples from the well-mixed material were used for cyanophycin analysis.

To extract plants to be implanted in the field, the seeds were planted in plates with blotting paper discs soaked in water to ensure appropriate emergence in Rosario (Argentina). Subsequently, the seeds were transplanted to seedling trays with GrowMix MultiPro soil (Terrafertil^®^) in normal watering conditions. Fertilization was performed by irrigation once each week with 2 g/L Hakaphos Green NPK 15/10/15. The photoperiod corresponded to 14 h light and 10 h dark. Two clippings were performed to each plant to stimulate root development, decrease leaf area and decrease the stress at the time of transplantation in the field.

### Field Cultivation

The seedlings were transferred from the greenhouse in Rosario to the Pergamino (Argentina) experimental field in February 2020. The materials were placed in 4 blocks with random distributions. Each plot consisted of 3 rows, 6 m long with 3 lines separated for 67 cm. In each row, 9 plants were planted; therefore, each plot had a size of 12 m^2^ and included 27 plants. The total trial size was 28 plots (6 events and NIC for 4 replicates). Fertilization was performed with urea (50 kg/ha) on the same day as the transplant and after 6 days. Surface drip irrigation was performed. To control insects, lambda-cyalothrin (Karate Zeón 150 cc/ha) was applied 30 days after transplantation. The following data were extracted in the field: fresh (fw) and dw of the bulk obtained from 18 plants from each plot, fw and dw of one plant from each plot, and fw and subsequent silage of two individual plants per plot. Harvesting was performed in May after 13 weeks in the field. Leaf material was air-dried or processed into silage as described and sent to Rostock. Before cyanophycin determination, the material was freeze-dried to make the dry weights comparable to the greenhouse data.

### Ensilage of Tobacco Plants

Whole tobacco leaves of transgenic BG and BG NICs were harvested and chopped to a particle size of 50 mm. Approximately 400 g was ensiled in vacuum-sealed polyethylene bags, with reference to (Hoedtke and Zeyner, 2011; Nausch et al., 2016). No volumetric measurements were performed for the control packing density or lactic acid bacterial content. Samples were stored at room temperature for several months (at least 2 months) before use.

### Germination and Segregation Assay

At least 150 seeds from self-fertilized transgenic plants were germinated on an LS medium containing 100 mg/ml kanamycin (Km), as described in (Hühns et al., 2008). Zygosity and the distinction between one and multiple integrations were determined by counting resistant seedlings. Notably, 75% Km-resistant seedlings were interpreted as one integration locus, and the case with any value greater than 80% was evaluated as an event with multiple integrations. Homozygosity was determined through the Km resistance in T2.

### CGP Isolation

The CGP isolation of different starting materials and controls was tested in duplicate in 30 ml extractions. The leaf material (1.5 g, dw) was mixed and homogenized with an Ultra Turrax (IKA T25, small shaft) at 25.000 rpm for three to 4 minutes in 30 ml 0.1 M HCl in a 50 ml conical Greiner tube. The pH was set below 1.3 before 30–60 min of extraction on a roller bank. The leaf material was separated by centrifugation for 20 min at 6,000 × g or 8,000 × g. The supernatant was decanted through two layers of Miracloth in another 50 ml tube. The pH was set to 4.0–4.5 with 5 M NaOH (1 M used for final setting) and mixed for 5 min to precipitate CGP. Subsequently, the extract was centrifuged for 20 min at 6,000 × g to pelletize CGP, and the supernatant was discarded. The CGP pellet was redissolved in 30 ml 0.1 M HCl with a potter tube. Either a 2 ml sample on SEC was directly measured, or the contamination was spun down through 20 min centrifugation at 9,000 × g. In another tube, CGP was precipitated again from the supernatant with 5 M NaOH at pH 4.0–4.5 and centrifuged for 20 min at 6,000 × g. The pellet was redissolved in 15 ml (to concentrate) or 30 ml 0.1 M HCl using a potter tube and placed undisturbed on a roller bank for 30 min to promote dissolution. A 2 ml sample was used for the SEC analysis. Extraction at pH 5 followed the same protocol, except homogenization was performed in 30 ml of HCl at pH 5, followed by acidification with 6 M HCl.

Dried leaf pretreatment: The dried samples contained up to 10 cm long leaf stems. One hundred grams of sample was mixed in a VitaPrep3 dry material beaker (VitaMix, Olmsted Falls, United States) for 2 minutes at low speed. Large pieces were manually crushed and separately mixed for 1 min. The complete sample was sieved through a 2 mm mesh, and the resulting 62–78% dw of the powder was used.

Silage pretreatment and extraction: To overcome the inhomogeneity of the silage material, extraction was performed at a larger scale, and the duplicates were split after mixing. A total of approximately 1,000 g silage (9.4% dw) was blended in a VitaPrep3 beaker (VitaMix, Olmsted Falls, United States) in 250 g portions with 500 ml of H_2_O per portion. The sample was acidified with concentrated HCl with pH = 1 and stirred for 1 h through a shaker. The sample was split into duplicate samples, each of which was centrifuged for 30 min at 3,000 × g. Subsequently, the abovementioned method was followed, except the extraction volume was 1 L instead of 30 ml.

### CGP Quantification

CGP analysis of field and greenhouse samples was conducted as described in (Hühns et al., 2008; Nausch et al., 2016) with certain modifications. Leaves were homogenized using a FastPrep beadmill (MP Biomedicals, Santa Ana, United States). A calibration curve was determined with CGP isolated from *E. coli* (Ziegler et al., 1998), with the CGP concentration ranging from 0.5–5 mg/ml. Bradford analysis of the pH = 1 and pH = 5 extraction samples was performed following a modified protocol for performing measurements in 96-well plates. Samples were diluted to a concentration of 1–∼8 μg/ml. The 5× RotiQuant reagent was diluted 2× with Milli-Q H_2_O and used at room temperature. Fifty microliters of the reagent was added to 200 µL of sample, and each well was mixed by 1× pipetting up and down. The absorption within 5 min was measured to be 595 nm by using a VersaMax microplate reader (Molecular Devices, San Jose, United States). The results were quantified using a calibration curve of 1–5 μg/ml CGP from tobacco leaves purified by 100 kD and 5 kD ultrafiltration.

CGP analysis of the isolation samples was performed through size exclusion chromatography over a Superdex75 10/300 GL column (GE, Chicago, United States) on an Akta Purifier 100 (GE, Chicago, United States). With a 200 µL injection loop and 0.6 ml/min concentration, 0.4 M H_3_PO_4_ + 0.3 M NaCl single eluents were treated at 25 ml. Detection was performed using RI and UV detectors at 220, 280 and 320 nm. Manual peak integration was performed on a UNICORN 7.5 device (classic mode), and the pure collected CGP fraction was calibrated using the calibration line defined as Peak area [mAu*mL] = 158.62*Concentration [mg/mL] + 2.6371 with *R* ^2^ = 0.99. Samples in 0.1 M HCl, with CGP ranging between 0.5 and 10 mg/ml, were centrifuged for 5 min at 20.000 × g before loading.

### Statistical Methods

Exploratory data analysis, the comparison of means and creation of box plots was carried out using IBM SPSS Statistics 22. Tests were chosen depending on the data properties. Normal distribution was tested using the Shapiro–Wilk test with *p* ≤ 0.05 defined as normally distributed. Homogeneity of variation was tested using the Levene statistic with *p* ≤ 0.05 defined as homogenous. Depending on these requirements and the respective dataset the corresponding statistical tests were chosen. A *p*-value ≤ 0.05 (two-sided) was considered significant.

To perform the cost–benefit analysis, Monte Carlo simulations involving 100,000 repetitions with triangular distributions were performed for extraction, purification and CGP prices for the extracted CGP and revenue. Table 2 presents the input data for the simulation. The extraction yield distribution and CGP price distribution were estimated. Since the purity requirements differed per application, for the purification distribution, the estimated min, max and mode values are used. The extraction yield distribution min and max values were obtained through experiments with dried leaves and silage.

In the choice-based conjoint (CBC) experiment, individual-level utility parameters were estimated using a hierarchical Bayesian (HB) multinomial logit model.

## Results

### Production of CGP in the Tobacco Variety Badischer Geudertheimer

The tobacco variety BG is bred and cultivated in Germany (Nicota GmbH, Rheinstetten, Germany). This variety provides high leaf and seed yields in the field and greenhouse and has already been used to produce CGP (Nausch et al., 2016).

**FIGURE 2:**
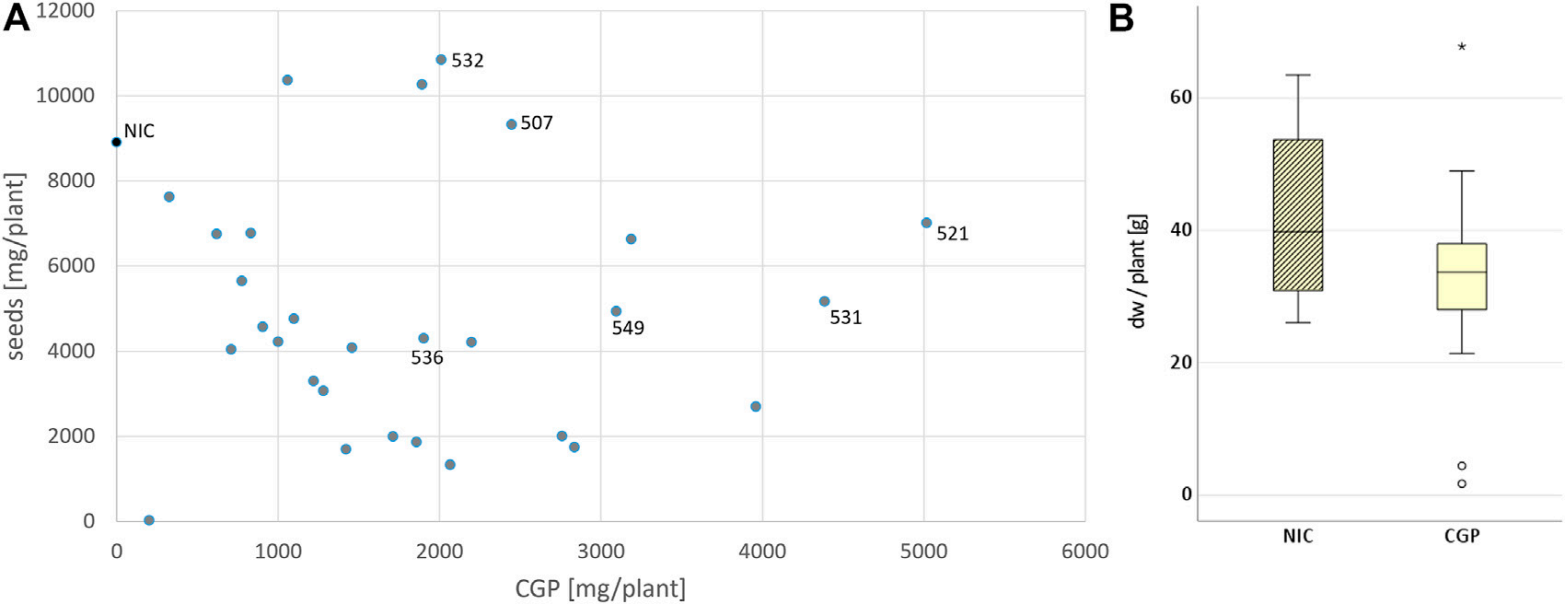
Influence of CGP production on the leaf and seed yield. **(A)** Distribution of the CGP and seed yield among all events and selected events. **(B)** Variation in the biomass (in dw/plant) in the NIC and all T0 events.

To produce plants with a high CGP content and without any yield penalty, BG was again transformed using the vector p*PsbY-CphA*_*Te*_ (Figure 1, (Hühns et al., 2008)). From the resulting transformants, 38 plants (T0) were tested *in vitro*. All events with a CGP yield lower than 1% dw *in vitro* were discarded. Leaf, seed and CGP productions in 31 events grown in the greenhouse were analyzed (Supplementary Table S1). Most of the events exhibited a slight, but not significant reduction in the leaf yield, while the seed yield was often drastically reduced compared to the NIC grown under the same conditions (Figure 2). Seed yield comparable to the NIC was obtained only for events with CGP contents of less than 3 g/plant (Figure 2). The plant phenotype was similar to the NIC and premature leaf senescence was observed (Supplementary Figure S2). The seed phenotype was normal. Six events (531, 521 with high, 507, 549 with medium, 532, 536 with low CGP dw) were selected for further analysis (Table 1). The number of integration loci in these events was determined via segregation analysis. Two events with the highest content corresponded to more than one loci (Table 1).

**TABLE 1:** T0 events selected for the field trial.

BG35SPsbY-cphA_TE_ event no	Biomass dw [g/Plant]	Seed Yield [g/Plant]	CGP/dw [%]	CGP/Plant [g]	Number of Integration Loci
531	38.6	5.2	11.4	4.4	>1
549	31.9	4.9	10.3	4.0	>1
521	67.8	7.0	7.4	5.0	1
507	41.2	9.3	5.9	2.4	1
536	38.9	4.3	4.9	1.9	1
532	44.1	10.8	4.6	2.0	1
NIC average (n = 6)	42.3 ± 14.7	8.1 ± 2.7	—	—	

### Performance of the T1 Generation in Field and Greenhouse Trials

**FIGURE 3:**
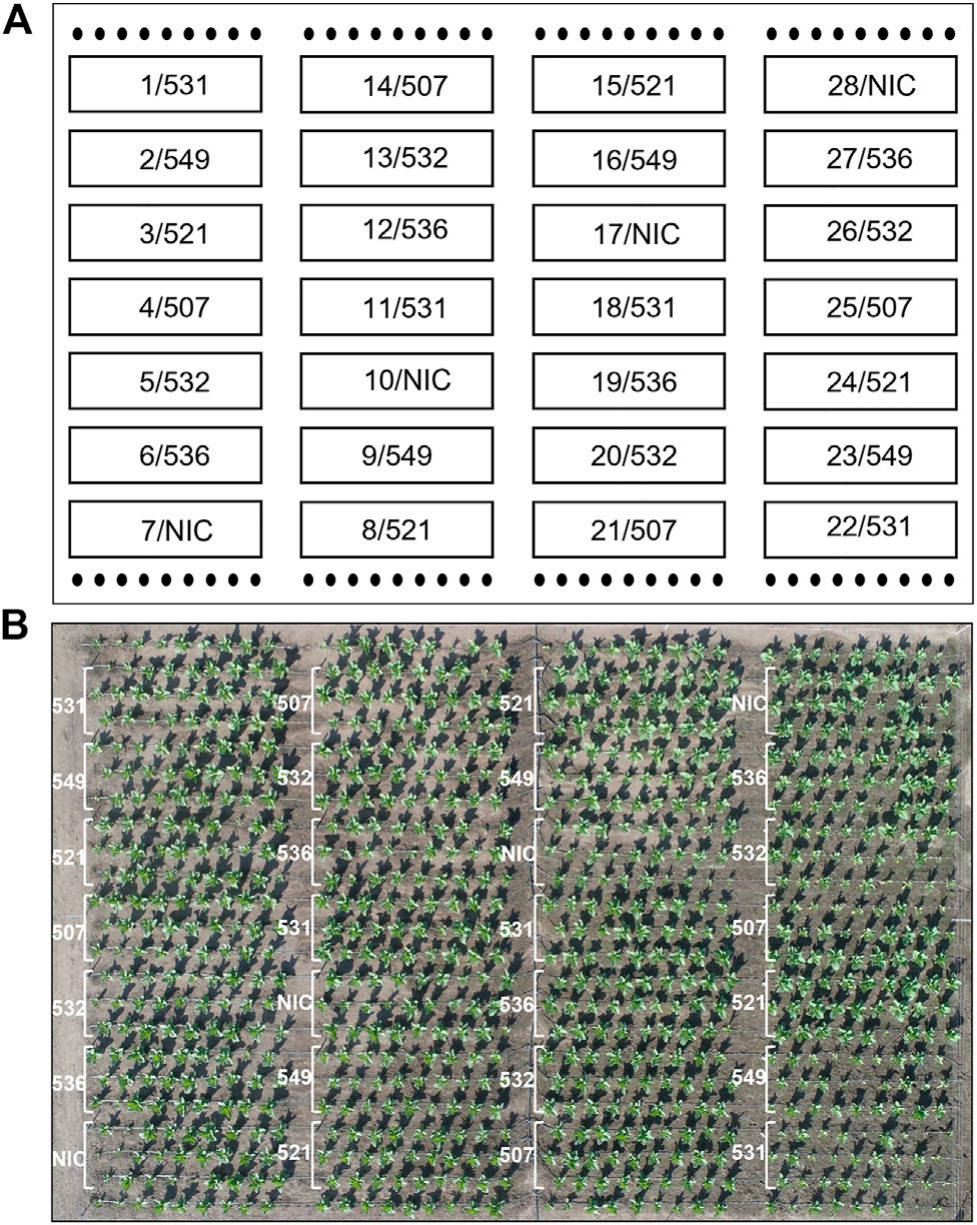
Field trial plan: 4 repetitions for each event, 3 rows and 9 plants per plot. **(A)** schematic field map; the numbers represent the plot and event;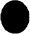 NIC plants as the border line. **(B)** Aerial image of the field 23 days after planting. White bars: plot borders.

**FIGURE 4:**
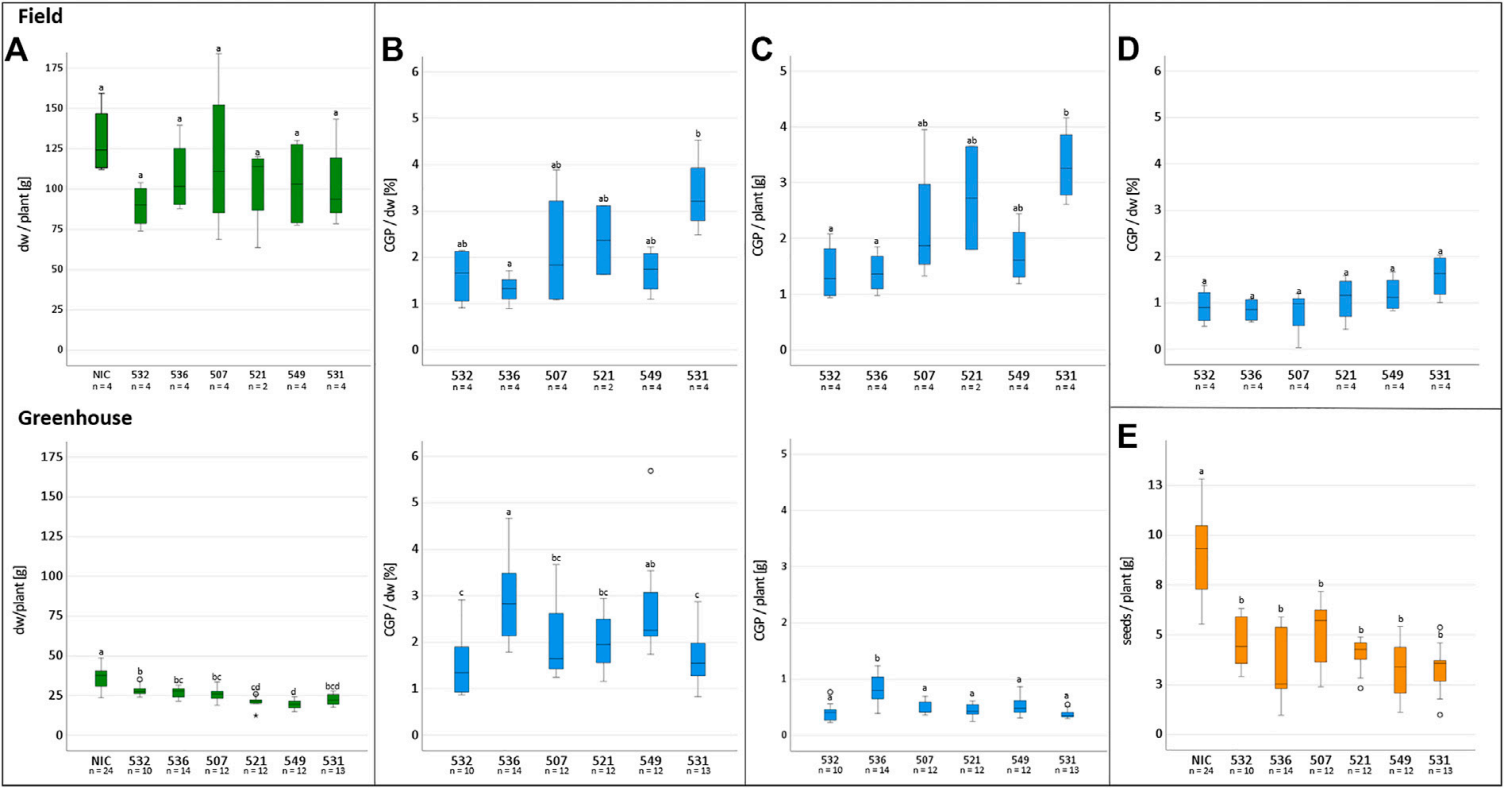
Influence of factors on the CGP production and tobacco biomass in field and greenhouse cultivation. Upper row: Field, lower row: greenhouse. **(A)** dw/plant, **(B)** CGP content/plant, **(C)** CGP content/dw, **(D)** CGP content/dw in bulk samples, **(E)** seed yield/plant in the greenhouse, **(A–D)** significance classes.

The T1 offspring of the six selected events were grown in Rostock (Germany) in a greenhouse and in parallel in Pergamino (Argentina) in the field. The phenotype, leaf biomass and seed yield of at least ten descendants/event were analyzed individually in the greenhouse. In the field, 27 plants/plot were planted in four repetitions (Figure 3). The phenotype of the transgenic plants was similar to that of the NIC, and drastic reductions in the plant growth seemed to depend more on the location than the event. For instance, significantly reduced growth was observed in the two bottom lines in plot 26 and two upper lines in plot 25, while the other plants of the events grew normally. The reduced growth observed in plot 22 for event 531 was not observed in the 531 repetitions. Similar results were obtained for the other events. One plant/plot was separately harvested, and the remaining entities were considered a bulk sample. Due to the massive regulatory burden, the T1 seeds arrived extremely late in Argentina; hence, planting was delayed, and seed production could not be realized. Leaf senescence did not occur prior to the harvest. The leaves of the greenhouse- and field-grown bulk material were either air dried or ensiled, while the single plants were stored as dried material. After determining the fresh and dry weight, the material was sent to Germany for further analysis. In contrast to the T0 generation, the average biomass of the transgenic plants was significantly reduced in the greenhouse but not in the field, compared to the NIC (Figure 4A). From each batch of dried leaves, three samples of approximately five to 10 g were randomly extracted, lyophilized and mixed thoroughly, and the final sample was subjected to CGP determination. The four single plants/event harvested separately in the field exhibited a significantly higher CGP content than the batch samples (Figures 4B,D). Since the offspring of hemizygous plants were analyzed, this phenomenon was attributable to the presence of zero segregates in the sample. These zero segregates also decreased the number of CGP-producing single plants in event 521. Therefore, we refer to the results obtained for the single plants in the following discussion. The best performer in the field, 531, exhibited a maximum CGP content of 4 g/plant (Figure 4C). The decrease in the CGP content/dw was similar in the field and greenhouse (Table 2). In the bulk samples, no significant difference was observed in the CGP production between the events (Figure 4D). For the single plants, the ranking of the events was different in the greenhouse and field. In the greenhouse, T1 of BG 536 was the best performer, while BG 521 and BG 531 exhibited extremely low CGP values. In contrast, in the field, BG 521 and BG 531 exhibited the highest CGP content/plant (Figure 4C). In the greenhouse, the seed yield was significantly decreased (Figure 4E).

**TABLE 2:** Calculated CGP production and revenue. The input values for the Monte Carlo simulation are underlined.

		Scenario	Min	Max	Mode
**Production**					
Planting density	D	plants/ha	22.000	22.000	22.000
CGP content	C	g/plant	4.0	4.0	4.0
CGP produced	PR = D*C	kg/ha	88	88	88
Extraction yield	EY		0.55	0.97	0.90
Purification yield	PY		0.70	0.93	0.90
Total yield factor	Y = EY*PY		0.39	0.90	0.81
CGP extracted	E = PR*Y	kg/ha	34	79	71
**Revenue**					
CGP price	P	USD/kg	275	550	330
CGP revenue (without simulation)	R = E*P	USD/ha	9.317	43.662	23.522

### Isolation From Dried Leaves and Silage

The laboratory isolation protocol reported for CGP isolation from CGP-engineered potato and tobacco (Neubauer et al., 2012) involves extensive maceration of lyophilized plant material using a homogenizer at pH = 1, centrifugation to separate the dissolved CGP from the solid plant material and precipitation of the CGP by neutralizing the extraction fluid to pH 5.0. We propose a more scalable and less energy-intensive process for CGP extraction from tobacco, using dried or ensiled tobacco leaves as the starting material. This process is more advantageous than the existing laboratory protocol because it is scalable, has already been applied to samples from field trials in Argentina, and requires considerably less energy because no lyophilization is involved.

Leaf material from the field trial was harvested as bulk per plot, mixed, partly ensiled and partly air-dried. In this manner, the CGP isolation from different forms of the starting material with the same initial CGP content could be compared. Silage was made by vacuum packing about 400 g chopped leaves and storing for minimum of 2 months. To overcome issues due to inhomogeneity of the silage material this extraction was performed at 1 L scale compared to 30 ml for the other extractions. It was confirmed that 1L and 30 ml extractions of the same material give equivalent results (S 3). Extractions were performed with a different pretreatment suitable for silage and dried leaves, followed by the same acid extraction at pH 1. To mimic milling of dried leaves in a lab setting, a dry blender was used. To simulate a refiner for silage treatment a blender was chosen as the best option. Silage was blended at pH = 5 before extraction. In an experiment on freeze dried leaf it was shown that maceration at pH = 1 or pH = 5 does not result in different yield of CGP (*p* = 0.05). Samples were extracted for 30–60 min in HCl of pH controlled below 1.3 before separation of liquid and leaf material by centrifugation and decanting. The extracted CGP was obtained by precipitation at pH 4.0–4.5 with NaOH, separated from the liquid by centrifugation. The extracted CGP was quantified by Bradford or size exclusion chromatochraphy and expressed as % CGP per dw of starting material.

Extraction efficiency is expressed as yield of a method or starting material (in %CGP per dw) compared to the yield (%CGP per dw) with the laboratory protocol. The standard laboratory protocol used lyophilized leaf powder as starting material, assuming the latter has the best CGP availability for extraction. Dried leaf showed an extraction efficiency of 55% (9%stdev, n = 2). Silage showed an extraction efficiency of 97% (13%stdev, n = 2) (Supplementary Figure S4).

## Cost–Benefit Analysis

Using the data pertaining to T0 and T1, we can calculate the expected CGP production per hectare. Since we selected elite events, the CGP content of the best performer (531) with a maximum CGP production of 4 g per plant was considered. The planting density of the trial was 22,000 plants per hectare, which is a standard value for high biomass tobacco production (Berbeć and Matyka, 2020). Hence, an extrapolated CGP production of 88 kg CGP per hectare was obtained. Since the extraction and purification yield and CGP prices involved uncertainties, we performed a Monte Carlo simulation. The input data for the simulation are presented in Table 2. Sallam and Steinbüchel (2010) reported that the industrial production cost of CGP from *E. coli* fermentation was between 250 and 500 EUR per kg CGP. We considered these values as references for the minimum CGP sales prices as crude material. Thus, these values were set as the min and max values of the CGP price distributions (P). The distribution results are summarized in Table 3, with the mean CGP production per hectare of 60 kg and a CGP revenue mean of 23.065 USD per hectare. Supplementary Figure S5 shows the revenue distribution.

**TABLE 3:** CGP yield per hectare distribution [kg/ha] and CGP revenue per hectare distribution [USD/ha].

	Min	1st Qu	Median	Mean	3rd Qu	Max
CGP extracted [kg/ha]	35.4	54.4	60.5	59.9	65.8	78.6
Revenue [USD/ha]	10,693	19,677	22,603	23,065	26,054	40,707

Moreover, Sallam and Steinbüchel (2010) predicted that a CGP dipeptide product would reach a market price of over 3,000 EUR per kg. If 3,300 USD per kg is considered the fine chemical CGP application price, a revenue of 198,000 USD per hectare can be expected in the fine chemical market for a CGP production per hectare of 60 kg. These calculated revenues show the margins for cultivation, isolation, purification and formulation costs. In other words, investments in extracting and processing CGP from tobacco plants for crude and fine chemical products can yield positive returns per hectare.

In 2018, biopolymer production reached 7.5 million tonnes (2% of the petrochemical polymer volume), and the compound annual growth rate of the biopolymer production is expected to be 4% until 2023 (Chinthapalli et al., 2019). The Coca Cola Company introduced the PlantBottle™, which contains 30% bio-PET, and 35 billion^1^ plant bottle packages were distributed between 2009 and 2015. Danone GmbH switched to a polylactic acid (PLA) version of the dairy cup to decrease the thickness of the cup wall for its Activia yogurt brand in Germany and Switzerland by 2011 (Schut, 2016). The car company Renault saved 0.40 EUR per part by switching to a biobased high heat ABS in the dashboard of Clio line (Roma, 2016). Farmers can lower their labor costs by switching to biodegradable plastic mulch film (van den Oever et al., 2017). These examples show that industries are seeking new biopolymers for different applications, and CGP is a promising candidate.

### Public Acceptance of CGP-Based Products

Representative studies of consumer reactions to CGP-based products in four European countries (Finland, Germany, Italy, Netherlands) indicate acceptance. We chose food wrapping material and skin cream as hypothetical applications requiring consumer acceptance and conducted choice-based conjoint (CBC) analyses. For food wrapping (N≈2680) , GM crop-based material was combined with antibacterial functionalities, environmental certification, and surcharge for wrapping. The options for wrapping material were conventional (petroleum-based, not decomposable), paper (recycled, partially compostable), GM bioplastic (derived from genetically modified tobacco plants as a byproduct, decomposable), and natural (waterproof plant leaves, to be imported). Each respondent was subjected to 12 choice situations, with each situation offering four wrapping alternatives (described with the attributes) and the “no wrapping” option. In all four countries, the utility parameters for GM bioplastic were positive and significant (95% confidence interval). On average, respondents indicated a higher utility for food wrapping made from *GM bioplastic* compared to *conventional plastic* (for more details, see Weisenfeld et al., 2022). Similarly, in terms of skin creams, in all countries (N≈2630) , creams based on *GM-oil* were preferred over conventional creams *based on petroleum*. The ingredient “oil” (petroleum, organic certified oil, GM-based oil) was combined with skin effects (Sun protection, moisturizing effect, antibacterial effects, none of these), certification (dermatologically tested, fair for life, ecofriendly or no certification), and price. Participants in all countries indicated positive and significant (95%) utility parameters for GM-based oil as a material.

## Discussion

The sustainable production of polymers is a relevant goal at present. Plants grown in the field provide an excellent platform for important compounds, especially when they are coproducts of traditional plant compounds such as oil, sugar or starch. Tobacco seems to be especially suited as a production platform for the following reasons: First, tobacco plants can be easily modified to produce specific compounds (Horsch et al., 1985), and second, the decline in smoking globally has imposed pressure on tobacco farmers (Jha and Chaloupka, 2000). Therefore, CGP production in leaves can provide an alternative income source for tobacco farmers. Nevertheless, it remains to be shown whether CGP production in tobacco is truly sustainable from ecological, economic and socioeconomic viewpoints. Several of these aspects were addressed in this paper.

One of the main factors influencing the extent of sustainability in production is the CGP yield/hectare. The average biomass of the CGP-producing plants in the field was not significantly decreased compared to that of the NIC. This finding is in agreement with (Schmidt et al., 2019), in which the production of a bacterial cellulase at 20% of the total soluble protein did not lead to a yield penalty. In contrast, CGP-producing potatoes were influenced in terms of the tuber size and yield in several field trials, even with considerably lower CGP contents (Schmidt et al., 2017). Hence, tobacco may be assumed to be appropriate for producing novel compounds and enabling higher production levels for transgene-encoded proteins compared to potato. This assumption was supported by the data obtained for the production of PHB in plants. Although the amount of PHB in the chloroplast of transgenic tobacco (0.4 mg/g dw) was lower than that in *Arabidopsis thaliana* (132 mg/g dw), it was considerably lower in potato (0,09 mg/g dw) (Bohmert et al., 2002).

The decrease in the seed yield observed in the greenhouse indicates that the production of CGP is an additional burden that, although insignificant for the leaf yield in optimal circumstances, may lead to decreased stress resilience of the transgenic plants. This aspect is also indicated by the decrease in the CGP content in the T1 generation in the field and greenhouse and might be caused by a selection of low CGP-producing cells to form seeds. This result is supported by the fact that the CGP production level was similar for all events in the bulk samples regardless of whether the T0 parent was a high or low producer (Figure 4D). Even the ranking of the expression levels between the six events was different among generations and growth conditions (field and greenhouse), indicating that the integration locus of the transgene is not the main limiting factor.

CGP production in the field was as high as that in the greenhouse in terms of the CGP percentage in the dry weight. Since tobacco plants produce significantly more biomass in the field, the CGP yield/plant was considerably higher in the field. Further field trials with seeds from homozygous best performers in the field must be performed to examine whether the CGP production can be increased. In addition, Pergamino (Argentina) is not a typical tobacco cultivation area. In conditions, for instance, those in Salta (Northern Argentina), in which tobacco is grown commercially, the stress may be decreased, and a higher biomass and decreased yield penalty may be achieved.

Other important factors for success are storage and extraction at a large scale. To date, CGP has only been extracted from lyophilized leaf material. The reported experiments show that CGP can be extracted from dried leaf and ensiled tobacco leaves. This observation proves that the storage of harvest is a feasible option. In this study, a blender was used to simulate the milling of dried leaves and maceration of silage. Since cell destruction, which requires the use of a refiner with steel components, can occur at pH = 5, corrosion of the steel must be drastically alleviated. The separation of CGP from the remaining plant material, which must occur at pH = 1, can occur in acid-resistant plastic containers. The difference in the CGP yield between dried leaf (55%) and silage extraction (97%) was considerable. This result may be attributed to the available CGP for extraction. Ensiling keeps the leaves wet, and microbial processes result in cell wall degradation. Moreover, ensiling decreases the pH through lactic acid production and increases the accessibility of cell components for extraction (Rooke and Hatfield, 2003). In contrast, CGP in dried leaves is less available for extraction, resulting in the lower extraction yield observed in this study. The availability of CGP in dried leaves can be enhanced by soaking dried leaves prior to extraction, milling the dried leaf to a powder form or increasing the extraction duration. The modifications introduced to the laboratory isolation protocol can also decrease the cost and facilitate the upscaling of this process.

The potential market for CGP-derived products remains to be validated. In addition to its potential for producing several different products, plant-made CGP is biobased and biodegradable and is thus more sustainable than most petroleum-based compounds. The advantages of biobased, biodegradable compounds have already been recognized by several companies.

Nevertheless, several technical and economic challenges remain in terms of decreasing the cultivation cost and efficiently upscaling the extraction while maintaining high extraction yields. At present, the extraction costs and yield at the hectare scale are unknown. This information can only be generated by establishing a pilot plant that can operate at the commercial scale. Such establishment requires upfront investment and political will to take risks. Farmers must modify their tobacco production strategies and may generate lower revenues for a certain period. Business models that moderate risk can help incentivize farmers and other parties to invest (Carraresi and Bröring, 2021). Nevertheless, in relation to the investments made in the EU for establishing biorefineries, the amount of investment required will be rather moderate and the risk will be relatively low (Clomburg et al., 2017). Policymakers can support development not only by supporting investments via subsidies but also by providing a stimulating policy environment (Smith et al., 2021). Cultivating transgenic tobacco plants and extracting and processing the CGP in Argentina appears to be a more cost-efficient strategy than that in which the tobacco is cultivated in Europe or Argentina and the crude CGP is exported to the EU, as indicated by recent research on the costs for approvals (Wesseler, et al., 2022 in press).

In addition, the decrease in the seed yield in the greenhouse may also occur in the field. This decrease may reduce the revenue of the primary product oil and influence the final revenue from the plants. This aspect must be analyzed in further field trials.

According to consumer choice experiments, on average, consumers reject petroleum-based products and favor natural or GM-based alternatives. These results indicate that consumer responses to GMO-derived products are context-sensitive. Environmental pollution has emerged as a notable concern of European citizens (Eurobarometer 19.1, 2019), and fossil fuel-based products and technologies are key causal factors. In situations in which consumers attempt to balance fossil fuel-based products and other options, GM-based alternatives gain acceptance. This observation might be suggestive of acceptance for products including CGP and its derivatives in Europe, which is important even if the product is not labeled.

## Concluding Remarks

This first field trial with CGP-producing tobacco showed that CGP production, storage of the leaf material and CGP extraction can be realized in the field on a larger scale without yield penalty. Further trials must be performed to examine whether the seed yield decreases in the field as in the greenhouse, and whether this decrease influences the usage of CGP as a coproduct. A purified CGP yield of 35–79 kg/ha can be obtained, which may be sufficient for commercial production; nevertheless, this yield depends on the final production and isolation costs. In addition, the CGP yield may change with the environment, planting date and year. Sallam and Steinbüchel mentioned the presence of a small market for CGP produced in microorganisms, and it remains to be seen whether possible new applications will further expand the market. Nevertheless, owing the high consumer acceptance for CGP products in even food-related applications, such as wrapping paper, CGP may emerge as a desirable raw material for companies.

## Data Availability

The original contributions presented in the study are included in the article/Supplementary Material, further inquiries can be directed to the corresponding author.

## Footnotes

1
https://money.cnn.com/2015/06/04/technology/coke-plastic-plant-bottle/

## Abbreviations

CGP, cyanophycin; BG: Badischer Geudertheimer; NIC, near isogenic control; CphATe, cyanophycin synthetase from Thermosynecchococcus elongatus BP-1; CphEPa, cyanophycinase from *Pseudomonas alcaligenes*; fw/dw, fresh weight/dry weight.
