# Corrigendum: Sustainable production of the cyanophycin biopolymer in tobacco in the greenhouse and field

**DOI:** 10.3389/fbioe.2022.985960

**Published:** 2022-08-29

**Authors:** Jana Huckauf, Boudewijn P. Brandt, Carlos Dezar, Henrik Nausch, Antoniya Hauerwaas, Ursula Weisenfeld, Ossama Elshiewy, Melina Rua, Jeroen Hugenholtz, Justus Wesseler, Kutay Cingiz, Inge Broer

**Affiliations:** ^1^ Agrobiotechnology, University of Rostock, Rostock, Germany; ^2^ Bioceres S.A., Rosario, Argentina; ^3^ Institute of Management and Organisation (IMO), Leuphana University Lüneburg, Lüneburg, Germany; ^4^ Wageningen Food and Biobased Research, Wageningen, Netherlands; ^5^ Agricultural Economics and Rural Policy, Wageningen University, Wageningen, Netherlands

**Keywords:** cyanophycin, plant made industrials, sustainable production, field trial, isolation, cost-benefit analysis, market analysis, consumer acceptance

In the published article, an **author name** was incorrectly written as “Aantoniya Hauerwaas”. The correct spelling is “Antoniya Hauerwaas”.

In addition, there was an error in the **Funding** statement. The correct Funding statement appears below:

Funding

This publication is part of the project Sustainable Co-Production [053.80.738] of the research programme [ERA-Net Cofund Action under the research and innovation programme Horizon 2020] “Tobacco as sustainable production platform of the natural biopolymer cyanophycin as co-product to oil and protein,” which is partly financed by the Dutch Research Council (NWO), the German Federal Ministry for education and research (BMBF) and by the Argentine government.

Finally, the **reference** for Weisenfeld, U. et al., 2022 was incorrectly written as Weisenfeld, U., Hauerwaas, A., Elshiewy, O., Halder, P., Wesseler, J., Cingiz, K., Broer, I. (2022). Beyond Plastic – a turning point for green biotechnology? Research Policy. It should appear as Weisenfeld, U., Hauerwaas, A., Elshiewy, O., Halder, P., Wesseler, J., Cingiz, K., et al. (2022). Beyond Plastic – a Turning Point for Green Biotechnology? Research paper, under Review.

The authors apologize for these errors and state that this does not change the scientific conclusions of the article in any way. The original article has been updated.

