# Supplementary material for: Sustainable Production of the Cyanophycin Biopolymer in Tobacco in the Greenhouse and Field
Source: Front Bioeng Biotechnol. 2022 Jun 13;10:896863. doi: 10.3389/fbioe.2022.896863 (PMC9234492; doi:10.3389/fbioe.2022.896863)
Supplement: Supplementary file 1 [file DataSheet1.docx]

Supplementary Material

**Supplementary 1.** Characterization of T0 transformands and the T1 offspring

| Event | dw [g] | CGP/dw [%] | CGP /plant [g] | seed/plant [g] | transgene integration loci |
| --- | --- | --- | --- | --- | --- |
| T0 green house | |  |  |  |  |
| 531 | 38.57 | 11.37 | 4.38 | 5.17 | ˃ 1 |
| 512 | 38.39 | 10.31 | 3.96 | 2.70 | 1 |
| 549 | 3.89 | 9.70 | 3.09 | 4.94 | ˃ 1 |
| 540 | 34.06 | 8.10 | 2.76 | 2.00 | ˃ 1 |
| 548 | 40.49 | 7.87 | 3.19 | 6.63 | 1 |
| 524 | 25.64 | 7.40 | 1.90 | 0 |  |
| 521 | 67.81 | 7.40 | 5.02 | 7.02 | 1 |
| 519 | 28.36 | 6.54 | 1.85 | 1.87 | ˃ 1 |
| 533 | 31.56 | 6.54 | 2.06 | 1.34 | ˃ 1 |
| 502 | 29.51 | 6.41 | 1.89 | 10.27 | 1 |
| 507 | 41.19 | 5.94 | 2.45 | 9.33 | 1 |
| 505 | 37.56 | 5.85 | 2.20 | 4.22 | ˃ 1 |
| 518 | 49.02 | 5.78 | 2.83 | 1.74 | ˃ 1 |
| 506 | 26.77 | 5.30 | 1.42 | 1.70 |  |
| 547 | 27.71 | 5.26 | 1.46 | 4.08 | 1 |
| 515 | 33.79 | 5.06 | 1.71 | 1.99 | 1 |
| 536 | 38.90 | 4.89 | 1.90 | 4.31 | 1 |
| 532 | 44.08 | 4.56 | 2.01 | 10.85 | 1 |
| 517 | 4.45 | 4.49 | 0.19 | 0.03 |  |
| 526 | 29.05 | 4.40 | 1.28 | 3.07 |  |
| 541 | 27.68 | 4.40 | 1.22 | 3.30 |  |
| 560 | 21.43 | 3.30 | 0.71 | 4.04 |  |
| 514 | 34.27 | 3.20 | 1.10 | 4.77 |  |
| 528 | 32.22 | 3.10 | 1.00 | 4.23 |  |
| 538 | 34.81 | 3.04 | 1.06 | 10.37 | ˃ 1 |
| 558 | 34.73 | 2.60 | 0.90 | 4.58 |  |
| 537 | 24.62 | 2.51 | 0.62 | 6.75 | ˃ 1 |
| 550 | 34.60 | 2.40 | 0.83 | 6.78 |  |
| 555 | 33.70 | 2.30 | 0.77 | 5.65 |  |
| 534 | 1.75 | 1.30 | 0.02 | 0.00 |  |
| 552 | 29.45 | 1.10 | 0.32 | 7.63 |  |
| NIC | 43.90 |  |  | 12.40 |  |
| NIC | 63.50 |  |  | 8.48 |  |
| NIC | 26.10 |  |  | 10.10 |  |
| NIC | 35.74 |  |  | 10.17 |  |
| T1 green house | |  |  |  |  |
| 532 | 35.24 | 0.87 | 0.31 | 5.91 |  |
|  | 26.24 | 0.89 | 0.23 | 6.32 |  |
|  | 29.44 | 0.93 | 0.27 | 5.20 |  |
|  | 27.74 | 0.99 | 0.27 | 6.15 |  |
|  | 34.04 | 1.08 | 0.37 | 3.81 |  |
|  | 28.70 | 1.62 | 0.46 | 4.64 |  |
|  | 27.30 | 1.64 | 0.45 | 2.91 |  |
|  | 24.34 | 1.9 | 0.46 | 4.19 |  |
|  | 24.04 | 2.32 | 0.56 | 3.41 |  |
|  | 26.34 | 2.91 | 0.77 | 3.56 |  |
| 536 | 21.64 | 1.79 | 0.39 | 5.90 |  |
|  | 22.54 | 1.98 | 0.45 | 5.38 |  |
|  | 31.34 | 2.12 | 0.66 | 2.16 |  |
|  | 30.44 | 2.14 | 0.65 | 2.52 |  |
|  | 29.30 | 2.51 | 0.73 | 5.85 |  |
|  | 24.14 | 2.55 | 0.61 | 5.89 |  |
|  | 28.64 | 2.76 | 0.79 | 2.31 |  |
|  | 28.20 | 2.89 | 0.81 | 3.93 |  |
|  | 27.54 | 2.98 | 0.82 | 2.33 |  |
|  | 31.50 | 3.34 | 1.05 | 1.73 |  |
|  | 24.54 | 3.48 | 0.84 | 0.98 |  |
|  | 29.40 | 4.23 | 1.24 | 3.62 |  |
|  | 23.54 | 4.43 | 1.04 | 2.57 |  |
|  | 26.60 | 4.67 | 1.24 | 2.49 |  |
| 507 | 33.54 | 1.25 | 0.42 | 5.64 |  |
|  | 26.14 | 1.38 | 0.36 | 6.16 |  |
|  | 25.24 | 1.43 | 0.36 | 6.26 |  |
|  | 29.54 | 1.43 | 0.42 | 6.33 |  |
|  | 26.14 | 1.54 | 0.40 | 4.24 |  |
|  | 25.84 | 1.61 | 0.42 | 2.40 |  |
|  | 29.34 | 1.68 | 0.49 | 7.17 |  |
|  | 23.74 | 1.78 | 0.42 | 6.25 |  |
|  | 26.14 | 2.40 | 0.63 | 5.81 |  |
|  | 19.74 | 2.85 | 0.56 | 3.32 |  |
|  | 22.64 | 2.99 | 0.68 | 3.93 |  |
|  | 18.94 | 3.67 | 0.69 | 3.35 |  |
| 521 | 25.94 | 1.16 | 0.35 | 4.01 |  |
|  | 25.74 | 1.36 | 0.40 | 4.61 |  |
|  | 20.94 | 1.54 | 0.38 | 4.62 |  |
|  | 20.24 | 1.58 | 0.39 | 4.28 |  |
|  | 22.50 | 1.81 | 0.45 | 4.89 |  |
|  | 12.40 | 1.91 | 0.25 | 4.02 |  |
|  | 22.24 | 1.99 | 0.52 | 4.25 |  |
|  | 21.84 | 2.02 | 0.58 | 2.35 |  |
|  | 20.24 | 2.34 | 0.55 | 4.45 |  |
|  | 20.80 | 2.66 | 0.61 | 3.54 |  |
|  |  | 2.70 |  | 4.67 |  |
|  |  | 2.94 |  | 2.84 |  |
| 549 | 17.94 | 1.74 | 0.31 | 4.12 |  |
|  | 21.74 | 1.80 | 0.39 | 5.12 |  |
|  | 19.6 | 2.07 | 0.40 | 3.44 |  |
|  | 24.24 | 2.20 | 0.53 | 5.42 |  |
|  | 21.64 | 2.21 | 0.49 | 4.00 |  |
|  | 17.24 | 2.25 | 0.43 | 1.12 |  |
|  | 21.1 | 2.27 | 0.62 | 3.36 |  |
|  | 15.00 | 2.52 | 0.48 | 2.30 |  |
|  | 19.20 | 2.94 | 0.68 | 4.64 |  |
|  | 15.24 | 3.20 | 0.87 | 1.95 |  |
|  |  | 3.54 |  | 1.82 |  |
|  |  | 5.69 |  | 2.22 |  |
| 531 | 27.84 | 0.83 | 0.32 | 3.00 |  |
|  | 25.70 | 0.84 | 0.33 | 2.69 |  |
|  | 26.34 | 1.16 | 0.34 | 3.57 |  |
|  | 24.54 | 1.28 | 0.37 | 3.71 |  |
|  | 21.44 | 1.28 | 0.33 | 2.85 |  |
|  | 18.04 | 1.51 | 0.29 | 3.66 |  |
|  | 2.90 | 1.55 | 0.39 | 5.38 |  |
|  | 20.74 | 1.64 | 0.41 | 4.55 |  |
|  | 19.70 | 1.72 | 0.55 | 4.60 |  |
|  | 17.60 | 1.98 | 0.50 | 2.56 |  |
|  |  | 2.23 |  | 1.79 |  |
|  |  | 2.81 |  | 3.70 |  |
|  |  | 2.87 |  | 0.99 |  |
| NIC | 38.24 |  |  | 6.61 |  |
|  | 40.44 |  |  | 8.59 |  |
|  | 37.64 |  |  | 7.20 |  |
|  | 43.54 |  |  | 10.18 |  |
|  | 40.84 |  |  | 9.07 |  |
|  | 41.04 |  |  | 10.43 |  |
|  | 38.54 |  |  | 7.31 |  |
|  | 48.74 |  |  | 5.55 |  |
|  | 38.94 |  |  | 6.59 |  |
|  | 29.74 |  |  | 7.27 |  |
|  | 28.64 |  |  | 10.08 |  |
|  | 32.14 |  |  | 7.76 |  |
|  | 33.30 |  |  | 12.44 |  |
|  | 23.64 |  |  | 12.35 |  |
|  | 32.90 |  |  | 12.24 |  |
|  | 23.64 |  |  | 9.34 |  |
|  | 37.60 |  |  | 12.84 |  |
|  | 46.40 |  |  | 10.30 |  |
|  | 34.10 |  |  | 10.54 |  |
| T1 field single plant | |  |  |  |  |
| NIC | 159.40 |  |  |  |  |
|  | 114.10 |  |  |  |  |
|  | 134.30 |  |  |  |  |
|  | 112.10 |  |  |  |  |
| 532 | 83.30 | 1.21 | 1.01 |  |  |
|  | 103.80 | 0.90 | 0.94 |  |  |
|  | 96.80 | 2.14 | 2.07 |  |  |
|  | 74.00 | 2.11 | 1.56 |  |  |
| 536 | 139.50 | 1.32 | 1.85 |  |  |
|  | 87.90 | 1.71 | 1.51 |  |  |
|  | 110.70 | 0.89 | 0.98 |  |  |
|  | 92.80 | 1.32 | 1.22 |  |  |
| 507 | 184.10 | 1.08 | 1.99 |  |  |
|  | 120.30 | 1.11 | 1.33 |  |  |
|  | 101.80 | 3.88 | 3.95 |  |  |
|  | 68.70 | 2.55 | 1.75 |  |  |
| 521 | 117.50 | 3.11 | 3.65 |  |  |
|  | 110.30 | 1.63 | 1.80 |  |  |
|  | 120.20 |  |  |  |  |
|  | 63.70 |  |  |  |  |
| 549 | 125.40 | 1.95 | 2.44 |  |  |
|  | 77.70 | 1.53 | 1.19 |  |  |
|  | 130.10 | 1.10 | 1.43 |  |  |
|  | 80.50 | 2.22 | 1.79 |  |  |
| 531 | 143.20 | 2.48 | 3.56 |  |  |
|  | 91.90 | 4.53 | 4.16 |  |  |
|  | 95.20 | 3.10 | 2.95 |  |  |
|  | 78.60 | 3.32 | 2.61 |  |  |
| T1 field bulk | |  |  |  |  |
| 532 |  | 0.73 |  |  |  |
|  |  | 0.49 |  |  |  |
|  |  | 1.38 |  |  |  |
|  |  | 1.07 |  |  |  |
| 536 |  | 0.67 |  |  |  |
|  |  | 1.08 |  |  |  |
|  |  | 0.58 |  |  |  |
|  |  | 1.04 |  |  |  |
| 507 |  | 1.19 |  |  |  |
|  |  | 0.97 |  |  |  |
|  |  | 0.98 |  |  |  |
|  |  | 0.03 |  |  |  |
| 521 |  | 1.34 |  |  |  |
|  |  | 0.42 |  |  |  |
|  |  | 1.58 |  |  |  |
|  |  | 0.97 |  |  |  |
| 549 |  | 0.92 |  |  |  |
|  |  | 1.30 |  |  |  |
|  |  | 0.83 |  |  |  |
|  |  | 1.66 |  |  |  |
| 531 |  | 1.01 |  |  |  |
|  |  | 1.90 |  |  |  |
|  |  | 2.02 |  |  |  |
|  |  | 1.36 |  |  |  |

**
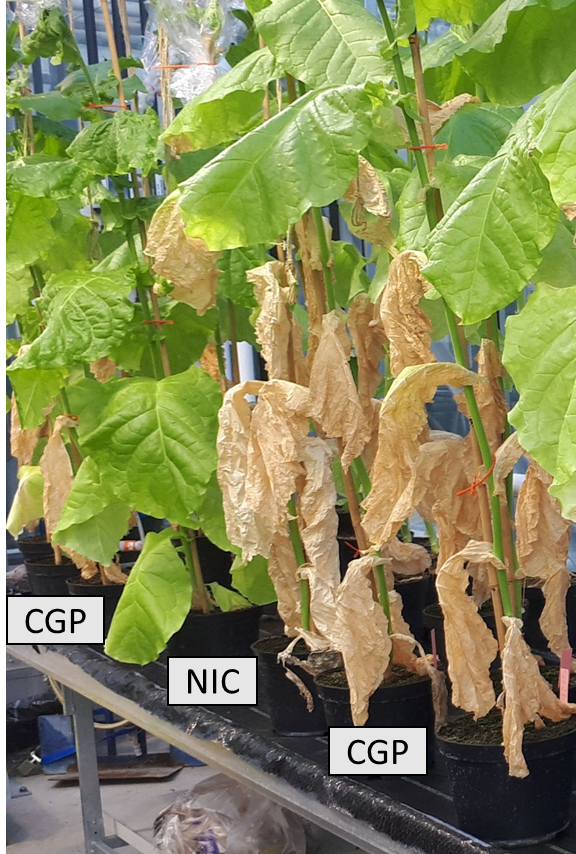
**

**Supplementary 2.** Premature leaf senescence in cyanophycin producing BG

**Supplementary 3.** Comparison of CGP yield from freeze dried leaves expressed as %CGP per dw, with 30 ml and 1l extraction volume in tree repetitions (A, B, C). (30 ml A was left out as outlier)

| **Volume** | **A % CGP dw** | **B % CGP dw** | **C % CGP dw** | **Average % CGP** | **% St dev** |
| --- | --- | --- | --- | --- | --- |
| 1 l | 3.15 | 3.25 | 3.18 | 3.19 | 1.71 |
| 30 ml |  | 3.26 | 3.32 | 3.29 | 1.42 |

**Supplementary 4.** Extraction yield from dried leaf, silage and lyophilized leaf from the same event to determine extraction efficiency.

| Event | state | Average CGP content [% DW] | % stDev | Extraction efficiency |
| --- | --- | --- | --- | --- |
| 507 | Dried | 1.36 | 0 | 55% |
| 507 | Silage | 2.39 | 9 | 97% |
| 507 | Lyopilized | 2.48 | 13 | defined 100% |
| NIC | Lyopilized | 0.00 | 110 |  |


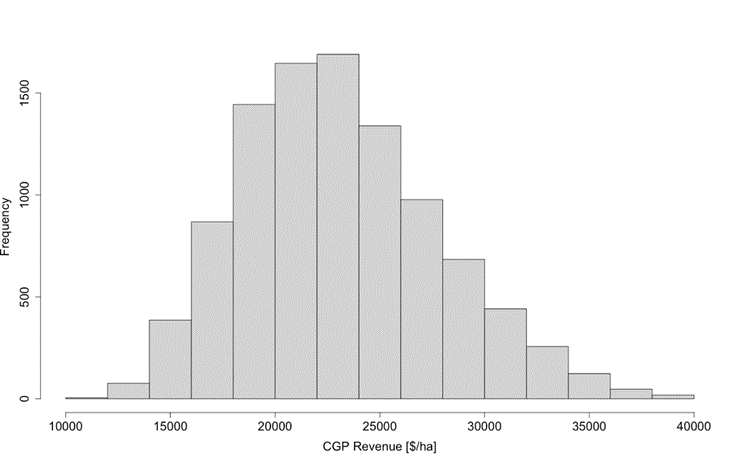


**Supplementary 5.** CGP Revenue per hectare distribution [USD/ha]
